# Changes in the Mandible Following Rapid Maxillary Expansion in Children with Class II Malocclusion: A Systematic Review

**DOI:** 10.3390/diagnostics12071688

**Published:** 2022-07-11

**Authors:** Małgorzata Kotarska, Nazan Kucukkeles, Joanna Lis, Beata Kawala, Kornelia Rumin, Michał Sarul

**Affiliations:** 1 Department of Integrated Dentistry, Wroclaw Medical University, 50-367 Wrocław, Poland; michal.sarul@umw.edu.pl; 2 Department of Orthodontics, Dental School, Bezmialem University, İstanbul 34093, Turkey; nkucukkeles@yahoo.com; 3 Department of Maxillofacial Orthopaedics and Orthodontics, Wroclaw Medical University, 50-367 Wroclaw, Poland; joanna.lis@umw.edu.pl (J.L.); beata.kawala@umw.edu.pl (B.K.); 4 Independent Researcher, 50-367 Wrocław, Poland; kornelia.rumin@gmail.com

**Keywords:** rapid maxillary expansion, class II, mandible, growing patient, systematic review

## Abstract

Objective: The aim of this systematic review was to determine whether rapid maxillary expansion (RME) allows favorable changes in the mandible during the treatment of class II malocclusion before the growth spurt. Methods: A search of Pubmed/Medline, the Science Direct, and the Google Scholar database was performed. The keywords used were: orthodontics, rapid maxillary expansion, class II, and growing patients. Relevant articles were assessed for quality according to Cochrane guidelines, and then changes in SNA, SNB, ANB, NL-NSL (or SN-SNP.SNA), and ML-NSL (or SN-Go-Gn) relationships were analyzed. Results: The selection process brought forth five articles, including 162 patients (91 females, 71 males) for detailed analysis. The quality of the evidence (GRADE) for comparisons and outcomes was assessed as moderate for SNB and ANB and as low or very low for other variables. Conclusions: The results of this systematic review showed that there is a small body of moderate-quality evidence for statistically and clinically favorable changes in SNB and ANB angles and a small body of low-quality evidence for changes in vertical parameters after RME.

## 1. Introduction and Objectives

Since Angell [1] introduced rapid maxillary expansion (RME) in 1860, regardless of continuous modification of the appliances used for this purpose, RME still involves separation of the maxillary midpalatal suture [2] in patients up to 15–18 years of age, who are prone to undergo this procedure [3,4,5]. As evidenced by meta-analyses, RME successfully corrects unilateral and bilateral crossbites and sagittal maxillary deficiencies, mainly in combination with maxillary protraction devices and/or a chin cap [6,7].

In addition to the objective evidence of RME effects on the maxilla [8], the literature also provides information about the beneficial RME effects on the mandible [9] (without overload of temporo-mandibular joints), which is expressed as a reduction in the facial vertical dimension and as a spontaneous class II correction [10,11].

Regardless of the scientific evidence for differences in RME achieved with bonded or banded hyrax screw [12], this issue is omitted in the literature, in either retrospective stud-ies [13], as well in systematic reviews [14] or meta-analyses [15].

Furthermore, notwithstanding a rigorous assessment of either the impartiality of the publications or correct reporting, the published evidence either omits the duration of an observation or admits the variety of additional (other than RME) interventions, which challenges the homogeneity of the study groups [16].

Considering new publications that have appeared in the literature since 2015 [17,18,19,20,21,22,23,24,25,26] including randomized clinical trials (RCTs) with potential evidence quality, as well as the previously published papers [27,28,29,30,31,32,33,34,35,36,37,38,39,40,41,42,43,44,45,46,47,48] requiring adequate data analysis, we designed our systematic review to reject two null hypotheses: 1. RME applied in growing patients does not induce stable vertical and sagittal changes that favor class II treatment; 2. the achieved changes do not depend on banding or bonding of HS.

## 2. Material and Methods

This systematic review was registered at PROSPERO (CRD42020184895). By performing the PRISMA protocol and following recommendations from the *Cochrane Handbook for Systematic Reviews of Interventions*, we defined the main research questions in PICO format (Table 1).

### 2.1. Search Strategy

**Figure 1:**
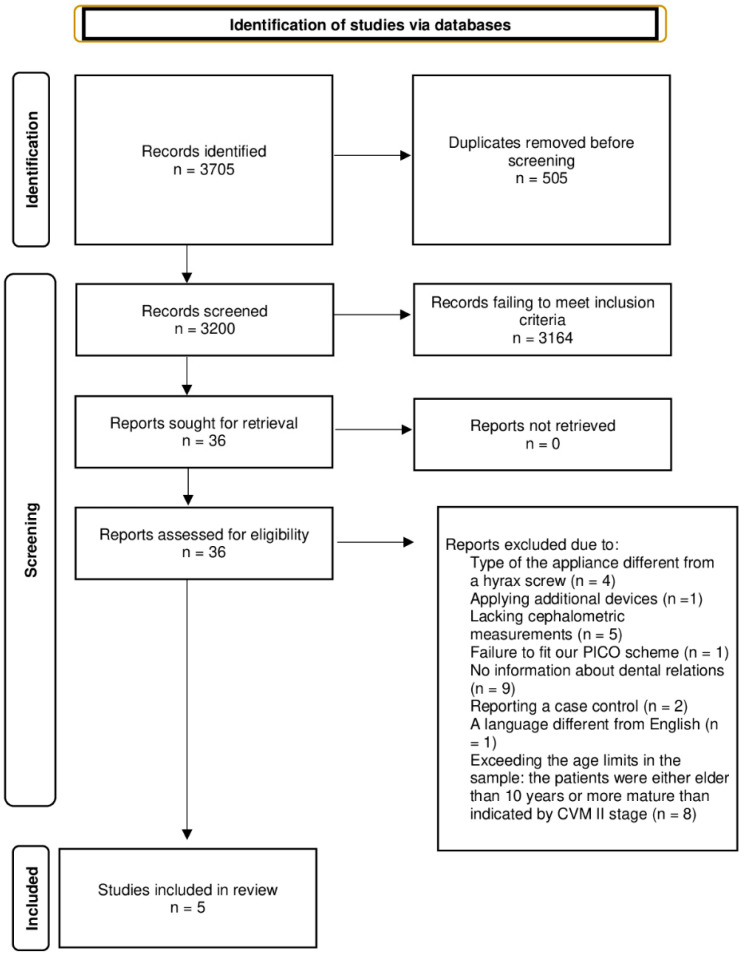
PRISMA diagram.

The search strategy of electronic databases Pubmed/Medline, Science Direct, and Google Scholar (1970 to December 2019) is shown in Table 2. The entire selection process is presented in the PRISMA diagram (Figure 1). 

### 2.2. Data Extraction

The remaining work was analyzed for suitability purposes within our study by two independent researchers (MK, KR). They downloaded the selected articles and contacted the authors of the papers if the data essential for our PICO format were missing. A lack of feedback either weakened a given article’s total score or excluded such a paper from our study. Experienced researchers (JL, BK, NK, and MS) independently verified the selection, and then the extracted data were compiled in a Microsoft Office Excel 2013 spreadsheet (Microsoft Corporation, Redmond, Washington, DC, USA); the level agreement was statistically evaluated with unweighted Cohen kappa statistics.

### 2.3. Quality Assessment

#### 2.3.1. Risk of Bias

The Cochrane Collaboration tool for assessing risk of bias in RCTs as low, unclear, or high enabled us to evaluate random sequence generation, allocation concealment, blinding of participants and personnel, blinding of assessors, incomplete outcome data, and selective reporting of outcomes.

The quality of the CCTs was assessed with a modified Newcastle–Ottawa Scale, composed of three sections:

1. Selection, evaluation of case definitions, representativeness of cases, control selection, and definition of controls. Each aspect was assigned 1 point, giving 4 points in total.
2. Comparability, appraising SNA, SNB, ANB, NL-NSL (or SN-SNP.SNA), and ML-NSL (or SN-Go-Gn) as the main parameters and observation time as an additional parameter, giving 2 points in total.
3. Outcome assessment, evaluation of outcome measures, duration of the follow-up, and blinding of assessors, giving 3 points in total.

#### 2.3.2. Evidence

We used the scale of the GRADE Working Group; thus, we evaluated the analyzed papers as high, moderate, low, or very low quality in terms of supporting the confidence in the estimate.

## 3. Results

The demographic structure of the pooled patient sample extracted from the articles, together with the ranges of sagittal and vertical changes in the mandible after RME application, is shown in Table 3. 

### 3.1. Quality Assessment

#### 3.1.1. Risk of Bias

The results of the risk of bias analysis in RCTs and in CCTs are presented in Table 4 and Table 5, respectively.

#### 3.1.2. Evidence

The quality of the evidence (GRADE) for comparisons and outcomes was moderate for SNB and ANB angles due to their statistically significant changes

Since an overall GRADE quality rating can be applied to a body of evidence across outcomes, usually by taking the lowest quality of evidence from all of the outcomes that are critical to decision making, we assessed our evidence as very low quality.

#### 3.1.3. Outcomes

Changes in the sagittal and vertical parameters, and their dependence on a different mode of HS mounting, are shown in Table 6. For the papers encompassing control groups, an inter-group comparison was carried out. The synthesis of results was in turn impossible due to the small sample sizes, insufficient data provided, or discrepancies between the results in the analyzed papers.

## 4. Discussion

There are reports in the literature regarding the rationale for using RME as an additional utility in class II treatment [33,42,49]. This rationale is based either on studies evaluating the morphology of the maxilla and the mandible, which turned out to be different in normal, distal, and mesial occlusions [50,51], or on reports regarding the lack of “self-correction” of distal occlusion during growth [52]. Improvements in the transverse dimension of the maxilla, which is narrowed in patients with class II [53,54,55,56], should facilitate both the protrusion and growth of the mandible, which fundamentally realizes the “foot to the shoe” concept [57]. This spontaneous class II correction, which is most often presented in studies evaluating dental relationships [33,58], is not supported by changes in the skeletal parameters analyzed in our study. Regarding the SNA angle, the body of *evidence* for all comparisons and outcomes was rated as very low quality. We found moderate-quality evidence for an increase in the SNB angle after 6 months of observation [30], suggesting the protrusion of the mandible, which follows the RME protocol. This study, however, involved children in whom changes in skeletal parameters resulted from growth; thus, a lack of comparison of the achieved changes with a control group challenges the reliability of the outcome. In turn, the comparable value of the SNB angle in the treated and control groups after a 4-year follow-up [34] may be related to the patients’ further development, namely, the CMS III phase, which is characterized by a significant increase in the mandible. A reduction in the ANB angle, also defined as a moderate quality of evidence, suggests both statistical and clinical improvements in class II malocclusion; nonetheless, it is the result of SNA changes and should also be treated with caution.

A statistically significant increase in the NL-NSL (or SN-SNP.SNA) angle, which is beneficial to the treatment of a class II open bite, occurred in one study [30], but had low-quality evidence caused by either the lack of a control group (fundamental in any research on growing patients) or the inability to compare the results with those of other studies. A statistically insignificant decrease in the ML-NSL (or SN-Go.Gn) angle in the one-year observation [20], clinically demonstrated as a counter-clockwise rotation of the mandible, regardless of a comparison with the control group, was assessed as evidence of a very low quality due to both the small number of patients and the discrepancies between the results.

A minor distal displacement of the mandible was the immediate effect of the banded RME. A decrease in the SNB angle may be related not only to the retraction of the mandible, but also to its clockwise rotation. In particular, a transverse overcorrection, i.e., raising the occlusion through contact of the palatal cusps of the maxillary teeth with the buccal cusps of their antagonists, resulted in a statistically significant increase in the ML-NSL (or SN-Go.Gn) angle in 28 patients [9]. This result, due to the occurrence in a single work and the small number of patients, was rated as very low quality evidence. On the other hand, the 6-month observation [30] theoretically allows us to conclude that the forward movement of the mandible is stable due to a statistically significant increase in the SNB angle and decrease in the ANB angle, regardless of the increase in the ML-NSL (or SN-Go.Gn) angle proving clockwise mandibular rotation. Nevertheless, due to the lack of comparison with the control group and due to the small number of studies and the patients analyzed, this information was considered as low-quality evidence.

There were discrepancies in the annual observations of the bonded HS effects [20,43]. Due to the minor changes in the SNA angle, the position of the maxilla was considered stable. A minor increase (comparing to control group) in the SNB angle allowed diagnosing a stable, statistically insignificant forward movement of the mandible. A statistically significant decrease in the ML-NSL (or SN-Go.Gn) angle, opposite to the increase recorded in the control group, allowed us to evaluate a clinically favorable counterclockwise rotation of the mandible [20]. Those outcomes were different to those reported by De Rossi et al. [43]; therefore, they were considered as very low quality evidence. A four-year follow-up [34] of the patients showed an insignificant decrease in the SNA angle after treatment, and its insignificant increase when compared with the control group. It brought low-quality evidence for a forward movement of the maxilla after RME application, compared to untreated patients. The lack of homogeneity of both the control and the intervention groups could have resulted in a comparable, but not statistically significant, increase in the SNB angle in the group treated with bonded HSs, as well as in the control group. It should be emphasized that the initial anterior–posterior position of the mandible was more favorable in the study group than in the control group, both at T1 and T3. The decrease in the ANB angle was twice as high in the study group than in the control group, but this change was statistically significant only in comparison with the initial situation.

As for the comparison of bonded and banded HS on the mandible, the differences in follow-ups presented do not allow for reliable evaluation, especially of the vertical changes. To do so, morphological changes in the mandible occurring post-RME treatment should be provided and thoroughly analyzed.

The body of evidence identified did not allow for an unequivocal determination of the skeletal changes after RME. Even if they were reproducible, these changes were assessed mainly on the basis of studies that were burdened with a medium risk of bias and involved only a few patients. Not all changes in the analyzed parameters could be compared with those in the control group, which made it difficult to draw conclusions. The works identified in this review also did not clearly determine the effect of the RME type on the mandible.

## 5. Potential Biases in the Review Process and Limitations

Any possible bias that could result from qualifying CCTs for this review was overcome using all possible methods for the objective selection of studies. An independent search, qualification for review, and risk of bias assessment were performed by two authors, and the dispute was resolved by an experienced researcher. Therefore, the main limitation of this work was the small number of qualified studies and the lack of representativeness of the groups. This is most likely due to the difficulty of achieving a homogeneous group of patients with only one form of intervention who were observed for an extended period of time during their growth.

## 6. Conclusions

Conclusions presented due to their insufficient value should be treated with caution. Based on this systematic review, the following can be suspected:

1. As there is a small body of moderate-quality evidence for changes in the SNB angle caused by the RME, regardless of its type, and because those changes are similar to the spontaneous ones occurring in untreated patients after a 4-year follow-up, the first null hypothesis is sustained.
2. Despite the fact that the effects of RME varied: counter-clockwise and clockwise rotation of the mandible following bonded and banded HS treatment, respectively, there is a small body of low-quality evidence; thus, the second null hypothesis is also sustained. Nevertheless, this systematic review could not include parameters demonstrating a changing morphology of the mandible, which are necessary for a reliable analysis and evaluation of the effect of bonded versus banded HSs on the mandible.
3. The lowest and close to the lowest grades of studies according to GRADE highly suggest the necessity of conducting thoroughly planned and reported studies of not only angular, but also morphological parameters in order to reject our null hypotheses. Due to ethical contraindications for creating and observing a control group, we suggest using control groups selected from existing growth studies.

## Figures and Tables

**Table 1:** PICO format.

Population	7- to 10-Year-Old Children with Stage I or II Cervical Vertebral Maturation, with Class II Malocclusion (3.5 < ANB < 6.0) Requiring Mandibular Advancement
Intervention	banded or bonded HSs prior to mandibular advancement
Comparision	no expansion preceding mandibular advancement
Outcome	rejection of the null hypotheses

**Table 2:** Search strategy.

Database	Key Words	Limits
PubMed/Medline	AND/OR: orthodontics rapid maxillary expansion rapid palatal expansion class II growing patients NOT: face mask	English language, studies on human, 1970—December 2019
Science Direct	AND/OR: orthodontics rapid maxillary expansion rapid palatal expansion class II growing patients NOT: face mask	English language, studies on human, 1970—December 2019
Google Scholar	AND/OR: orthodontics rapid maxillary expansion rapid palatal expansion class II growing patients NOT: face mask	English language, studies on human, 1970—December 2019

**Table 3:** The composition of the study material.

		Pereira [17]	Lione Intervention Group [20]	Lione Control Group [20]	Farronato [30]	De Rossi [43]	Guest Intervention Group [34]	Guest Control Group [34]
**Year of publication**		2017	2017	2017	2011	2011	2010	2010
**Female (n)**		16	5	5	28	14	31	22
**Male (n)**		12	5	5	27	12	19	28
**Age**		8.18 SD 0.9	8.1 SD 0.6	8.0 SD 0.8	8.8 SD 1.4	8.7 SD 1.5	8.8 SD 1.1	8.9 SD 0.9
**Skeletal** **maturity**		NR	CS1-CS2	CS1-CS2	NR	NR	Pre-pubertal at the beginning and post-pubertal at the end	Pre-pubertal at the beginning and post-pubertal at the end
**Observation intervals**	**T1–T2**	3 weeks	NR	NR	NR	NR	NR	NR
	**T1–T3**		1 year	1 year	6 months	12.2 months	4 years	4 years
**Width of the maxilla**	**T1**	transverse discrepancy, dentoalveolar width = 53.08 mm	negative posterior transverse interarch discrepancy ≥ 4 mm	negative posterior transverse interarch discrepancy ≥ 4 mm	transverse hypoplasia and presence of bilateral posterior cross-bite	posterior cross-bite combined with narrowing of the maxilla	constriction: initial mean transpalatal width = 30 mm or less	constriction: initial mean transpalatal width = 30 mm or less
	**T2**	Dentoalveolar width =+5.03 mm SD 1.66	NR	NR	NR	NR	NR	NR
	**T3**	NR	“until the palatal cusps of the maxillary posterior teeth approximated the lingual cusps of the mandibular posterior teeth”	NR	over-correction, not specified in numbers	“occlusal inclines on the palatal cusps of the upper molars occluded with the occlusal inclines of the buccal cusps of the lower molars”	“until the palatal cusps of the maxillary posterior teeth approximated the lingual cusps of the mandibular posterior teeth”	NR
**Hyrax screw mounting**		banded tooth-tissue-born	bonded	no intervention	banded tooth-born	bonded	bonded	no intervention
**SNA (°)**	**T1**	81.5 SD 3.04	79.2 SD 3.5	82.2 SD 3.7	79.88 SD 1.79	80.42 SD 4.54	81.3 SD 3.5	80.4 SD 3.0
	**T2**	0.41 SD 1.40	NR	NR	NR	NR	NR	NR
	**T3**		0.0 SD 0.4	−0.2 SD 0.9	80.32 SD 1.63	79.96 SD 3.93	0.3 SD 1.6	0.7 SD 1.8
**SNB (°)**	**T1**	77.29 SD 3.27	74.4 SD 3.6	76.4 SD 3.8	74.02 SD 2.08	76.88 SD 4.99	76.2 SD 3.5	74.9 SD 2.6
	**T2**	−0.47 SD 1.33	NR	NR	NR	NR	NR	NR
	**T3**	NR	0.5 SD 0.5	0.2 SD 0.7	76.27 SD 1.79	76.73 SD 4.71	1.2 SD 1.4	1.1 SD 1.6
**ANB (°)**	**T1**	4.14 SD 2.97	4.6 SD 1.2	5.8 SD 1.5	5.86 SD 1.03	3.53 SD 2.43	5.1 SD 1.8	5.4 SD 2.1
	**T2**	0.89 SD 2.20						
	**T3**		−0.7 SD 0.6	−0.3 SD 1.0	4.05 SD 1.28	3.23 SD 2.86	−0.9 SD 1.3	−0.4 SD 1.4
**NL-NSL (or SN-SNP.SNA) (°)**	**T1**	NR	NR	NR	9.65 SD 1.75	NR	NR	NR
	**T2**	NR	NR	NR	NR	NR	NR	NR
	**T3**		NR	NR	10.62 SD 1.68	NR	NR	NR
**ML-NSL (or SN-GO.GN) (°)**	**T1**	34.81 SD 5.08	33.8 SD 4.3	30.7 SD 5.2	32.41 SD 3.34	37.40 SD 5.82	NR	NR
	**T2**	1.80 SD 1.91	NR	NR	NR	NR	NR	NR
	**T3**	NR	−0.6 SD 0.8	0.9 SD 0.7	33.2 SD 1.68	37.59 SD 5.49	NR	NR

Legend: n—the number of patients; T1—the beginning of treatment; T2—immediately after expansion of the maxilla; T3—after treatment.

**Table 4:** Assessment of the risk of bias in RCTs.

Study	Random Sequence Generation	Allocation Concealment	Blinding of Participants and Personnel	Blinding of Outcome Assessment	Incomplete Outcome Data	Selective Reporting
Pereira et al., 2017 [17]	Low	Low	Low	Low	Low	Unclear
Lione et al., 2017 [20]	Unclear	Low	Low	Low	Low	Low

**Table 5:** Assessment of the risk of bias in the CCTs.

Study	Selection	Comparability	Outcome
Farronato et al., 2011 [30]	**	**	**
De Rossi et al., 2011 [43]	**	*	**
Guest et al., 2008 [34]	***	*	**

* 1 point; ** 2 points; *** 3 points.

**Table 6:** Characteristics of post-RME changes in the sagittal and the vertical parameter values.

	Pereira	Lione ^c^	Lione ^i^	Farronato	De Rossi	Guest ^c^	Guest ^i^
The follow-up (months)	0	12	12	6	12	48	48
HS mounting mode	Ba	Bo	Bo	Ba	Bo	Ba	Ba
SNA (°)	+0.41	+0.2	0.0	+0.44	−0.46	−0.4	+0.3
SNB (°)	−0.47	+0.3	+0.5	+2.25 *	−0.15	+0.1	+1.2 *
ANB (°)	+0.89	−0.4	−0.7	−1.81 *	−0.3	−0.5 *	−0.9 *
NL-NSL (or SN-SNP.SNA) (°)	NR	NR	NR	+0.97 *	NR	NR	NR
ML-NSL (or SN-Go.Gn) (°)	+1.8 *	−1.5 *	−0.6	+0.79	+0.19	NR	NR

*: statistical significance (*p* < 0.05); ^c^: comparison with control group; ^i^: comparison with initial measurements; the follow-up: post-RME observation period; Bo—bonded; Ba—banded; “+”: an increase; “−“: a decrease; NR: not reported.

## Data Availability

Not applicable.
